# Observation and Control of Gene Expression Noise: Barrier Crossing Analogies Between Drug Resistance and Metastasis

**DOI:** 10.3389/fgene.2020.586726

**Published:** 2020-10-30

**Authors:** Michael Tyler Guinn, Yiming Wan, Sarah Levovitz, Dongbo Yang, Marsha R. Rosner, Gábor Balázsi

**Affiliations:** ^1^Biomedical Engineering Department, Stony Brook University, Stony Brook, NY, United States; ^2^Laufer Center for Physical and Quantitative Biology, Stony Brook University, Stony Brook, NY, United States; ^3^Stony Brook Medical Scientist Training Program, Stony Brook, NY, United States; ^4^Ben May Department for Cancer Research, The University of Chicago, Chicago, IL, United States

**Keywords:** synthetic biology, non-genetic heterogeneity, noise, control, metastasis, cancer, drug resistance

## Introduction

Cancer metastasis is still the main cause of death for most cancer types (Dillekås et al., 2019). The molecular causes of metastasis are diverse, complex, and poorly understood, including genetic and other molecular changes that transcend genetic sequence. Despite their complexity and diversity, a new emerging theme posits these changes generate cellular heterogeneity that can promote cancer metastasis (Fidler, 1978; Lee et al., 2014; Nguyen et al., 2016; Caswell and Swanton, 2017). Cellular heterogeneity can be genetic or non-genetic. Besides genetic mutations, non-genetic heterogeneity allows otherwise identical cells to develop drastically different phenotypes due to variations in molecular players that accumulate and compound effects over time.

How could cellular heterogeneity affect metastasis? A successfully metastasizing cell must cross multiple physical and molecular barriers: it must detach from the primary site, intravasate, survive the bloodstream or lymphatic vessels, extravasate, overcome immune attack, and start growing. Therefore, understanding how cellular heterogeneity affects barrier crossing is quintessential to understand its role in metastasis. Moreover, other barrier-crossing phenomena, such as drug resistance, may be unexpectedly informative, or even analogous to various steps in metastasis.

Non-genetic heterogeneity and biological noise are broad terms we consider synonymous here. They include variation in essentially any cellular property that is not genetic in origin, such as cell size, protein levels, cell function, and lifespan. A subtype of non-genetic heterogeneity relevant to this article is gene expression noise, which manifests as varying messenger RNA (mRNA) or protein levels in cells with identical genomes. While there are different uses of the term “noise” across the fields of biology, we define noise here as in physics and engineering disciplines besides biology: as a general stochastic process that does not exclude heritability. Indeed, the existence of noise with various spectra (1/f noise, colored noise, etc.) in nature implies that there can be various short- and long-term components of randomness.

Understanding the role of gene expression noise in metastasis can be based on two complementary investigative approaches, which are conceptual generalizations of forward and reverse genetics (Gurumurthy et al., 2016), respectively. Namely, *forward (observational) investigation* (Schuh et al., 2020; Shaffer et al., 2020) monitors and catalogs naturally occurring gene expression variability at various molecular levels in different cell types, seeking associations with cancer progression from an observational perspective. By contrast, *reverse (perturbational) investigation* (Kang et al., 2013; Nguyen et al., 2016) studies cancerous phenotypes arising upon artificially imposed gene expression noise, using noise-controlling genetic devices, methods or chemicals (Desai et al., 2020). Here, we discuss both perspectives regarding gene expression noise and metastasis and outline how the reverse approach may be necessary due to a natural coupling between the noise and mean of gene expression, and how it may be accomplished through synthetic biology.

## Non-Genetic Heterogeneity and Threshold Crossing in Cellular and Molecular Processes

Genetic heterogeneity among cells, tissues, and organisms has long been known to play roles in generating the phenotypic diversity that life exhibits (Nichol et al., 2019). Genetic heterogeneity can cause two main types of clonal variation in a cell population (Agozzino et al., 2020). First, coding sequence mutations generate clonal populations with protein molecules of missing or altered function (e.g., a mutant enzyme loses or improves its affinity for a substrate). Second, changes in non-coding sequence or gene copy number create clones with altered mRNA or protein levels, without any changes in protein function (e.g., the amount of unaltered enzyme molecules increases or decreases). This second type of genetic heterogeneity resembles non-genetic heterogeneity or gene expression noise, especially if the latter is heritable (i.e., cellular memory is long) (Acar et al., 2005; Nevozhay et al., 2012; Shaffer et al., 2020), since they both manifest as lasting and propagating cell–cell differences in the number of protein or RNA molecules. Indeed, non-genetic variability, like genetic mutations, can play important roles in physiological processes, disease development, and evolution (Brock et al., 2009; Balázsi et al., 2011; Chattwood and Thompson, 2011; Frank and Rosner, 2012; Bai et al., 2013).

Two key characteristics of gene expression noise are its amplitude and its memory. First, the amplitude of gene expression noise [measured by the standard deviation or coefficient of variation (CV)] characterizes how far molecule numbers can deviate from the mean. Noise amplitudes can range from slight (CV < 20%) to dramatic (CV > 200%), giving rise to vastly different cellular phenotypes (Kærn et al., 2005; Raj and van Oudenaarden, 2008). Second, the cellular memory of gene expression noise characterizes the heritable aspects of random cellular differences. Cellular memory is, at least conceptually, independent from the noise amplitude (Acar et al., 2005; Nevozhay et al., 2012) and can range from <1 cell cycle time to hundreds of cell generations (Nevozhay et al., 2012), enabling a non-genetic version of clonal expansion that affects genetic evolutionary dynamics (González et al., 2015; Bódi et al., 2017; Kheir Gouda et al., 2019).

Gene expression noise can have many sources, including biochemical reactions of transcription, translation, post-translational modifications, mRNA/protein degradation, and other cellular processes (Kærn et al., 2005; Balázsi et al., 2011). These processes affect biological noise, which can be segmented as “intrinsic” or “extrinsic” (Thattai and van Oudenaarden, 2001; Elowitz et al., 2002; Swain et al., 2002). Intrinsic noise comprises variation intrinsic to gene expression due to stochastic effects from biochemical reactions involving low copy numbers of molecular species along the central dogma of molecular biology (Ozbudak et al., 2002; Swain et al., 2002; Quarton et al., 2020). This contrasts with extrinsic noise that describes variations in more global factors affecting gene expression, such as global regulators, ribosomes, polymerases, cofactor concentrations, microenvironmental variation, and activity of other cellular players extrinsic to the gene (Swain et al., 2002; Stamatakis et al., 2011). In addition, non-genetic heterogeneity (noise) in protein levels can be preexisting or induced. Unlike tightly regulated deterministic stress programs where most cells turn on the same stress response, induced heterogeneity implies random cell–cell differences emerging upon exposure to stress (Gasch et al., 2017; Farquhar et al., 2019), where some cells turn on various forms of stress response while some other cells do not.

**Figure 1:**
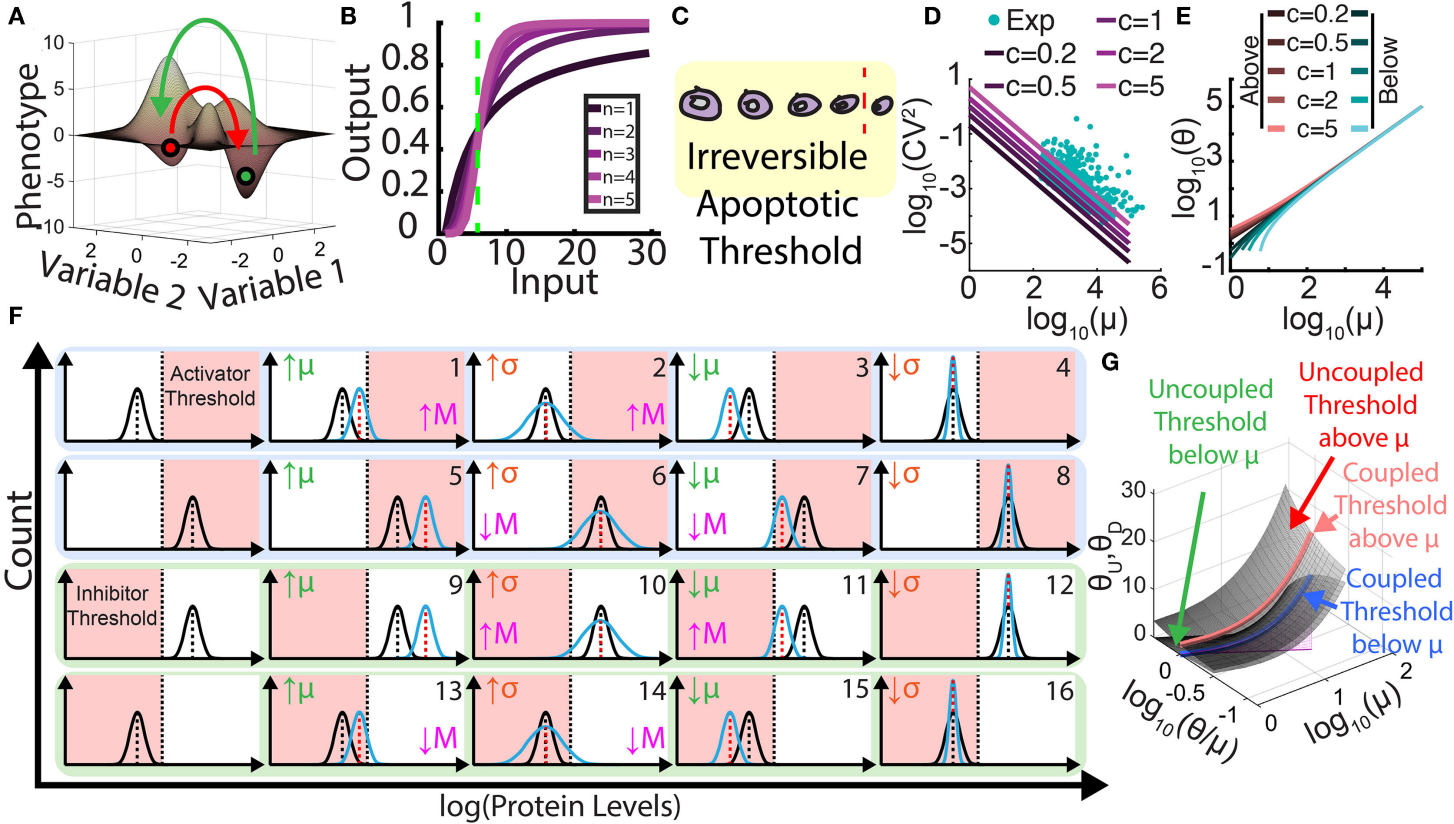
Role of heterogeneity and its control in threshold crossing and metastasis. **(A)** Multistable landscape illustrating two steady states (phenotypes). Red arrow represents going from the first phenotype to the second across the crest (threshold), and the green arrow represents the reverse transition. **(B)** Ultrasensitivity is the abrupt change in output for small input changes, captured by Hill functions with high Hill coefficients, *n* ≥ 2. Green line represents threshold where the system is most sensitive to input. **(C)** Irreversibility stems from a physical or chemical threshold that acts as a point of no return when cells surpass it. **(D)** Graph illustrating the natural tendency of gene expression noise [coefficient of variation squared (CV^2^)] to decrease hyperbolically as mean levels increase: CV^2^ = *c*/mean. The values of *c* ≠ 1 correspond to non-Poisson behaviors, such as bursting (*c* > 1) or noise suppression (*c* < 1, e.g., by negative feedback). Teal scatter dots represent rescaled yeast experimental data (Newman et al., 2006) illustrating the natural tendency of coupling between the CV and the mean for hundreds of genes. **(E)** Graph of threshold levels θ that can be crossed as a function of mean protein levels μ, with natural mean-noise coupling for bursting parameter *c*. Orange colors represent downward threshold crossing (heuristically, θ < μ – σ) when the mean is above the threshold, whereas the blue colors represent upward threshold crossing (θ > μ + σ) when the mean is below the threshold. **(F)** Threshold crossing effects of independently changing the mean μ or standard deviation σ for four starting positions of cell populations relative to phenotypic thresholds. The graphs with blue background (1–8) relate to metastatic activator levels, while the graphs with green background (9–16) relate to metastatic inhibitor levels. Pink areas represent metastasis (M), while white areas represent no metastasis, with pink arrows indicating increased or decreased likelihood. **(G)** Landscapes and curves illustrating phenotypic threshold levels that can be overcome (crossed) when the noise (CV) and mean (μ) vary in a coupled or uncoupled manner, respectively. The higher surface corresponds to upward threshold crossing [top and bottom rows of **(F)**, 1–4 and 13–16], while the lower surface corresponds to downward threshold crossing [middle rows of **(F)**, 5–8, 9–12 ]. Thresholds crossed by naturally coupled mean and noise values are overlaid on each surface as blue and orange lines.

Both intrinsic and extrinsic noise can affect developmental and evolutionary processes. Underlying such effects are threshold-crossing cellular processes that noise can promote or suppress. Noise of drug resistance protein levels can promote microbial and cancer cell populations to cross survival thresholds and thus resist high levels of drug treatment (Blake et al., 2006; Brock et al., 2009; Fraser et al., 2009; Shaffer et al., 2017). On the other hand, noise can also hinder short-term cell survival at low levels of drug treatment (Blake et al., 2006). In addition, recent evidence indicates that preexisting or stress-induced noise can play similar dichotomous roles during long-term evolution of cell populations (Fraser et al., 2009; Marusyk et al., 2012; Farquhar et al., 2019). While less established, there are suggestions that protein noise promotes oncogenesis (Brock et al., 2009), epithelial-to-mesenchymal transition (EMT), mesenchymal–epithelial transition (MET), and the initiation of metastasis (Lee et al., 2014; Nguyen et al., 2016; George et al., 2017; Jolly and Celià-Terrassa, 2019). These findings provoke the question: Could there be common principles of non-genetic heterogeneity underlying all these biologically different, but mechanistically similar processes? For example, higher, heritable noise can cause phenotypic changes in more members of a cell population by pushing and keeping cells above thresholds (Charlebois et al., 2011) that arise from multiple sources (Figure 1A), such as multistability, hypersensitivity, and irreversibility, which we discuss next.

Multistability is the property of a system to permit multiple potential steady states (Gardner et al., 2000; Macía et al., 2009) (two at the minimum), such as restraint vs. commitment to sugar utilization (Novick and Weiner, 1957), oocyte maturation (Xiong and Ferrell, 2003), and stem cell differentiation (Macarthur et al., 2009), which could be imagined as valleys in a landscape (Yuan et al., 2017; Kang et al., 2019; Agozzino et al., 2020) (Figure 1A). Stability of any steady state implies that effects of small, temporary external perturbations decay over time, so cells will return to their valley bottoms after weak noise or transient environmental fluctuations push them slightly away. However, sufficiently large temporary perturbations can alter protein means or noise to a degree that moves cells uniformly or individually beyond the crest (separatrix) separating two valleys, causing them to fall into the neighboring valley. One common theme underlying natural and engineered multistability is positive feedback embedded in biomolecular networks (Gardner et al., 2000; Angeli et al., 2004; Nevozhay et al., 2012), which enable cell survival and resistance to various environmental stresses (Charlebois et al., 2011; Farquhar et al., 2019) and cancer cell transitions (Lee et al., 2014). Two examples of barrier crossing while switching steady states may be EMT and MET, which are vital processes in embryonic development, tissue repair, and cancer metastasis (Zhang et al., 2014; Nieto et al., 2016; Jolly et al., 2017, 2018, 2019; Li and Balazsi, 2018; Gómez Tejeda Zañudo et al., 2019). However, it is increasingly accepted that the phenotypic spectrum between epithelial (E) and mesenchymal (M) cell states includes one or more intermediate states, so noise may induce such intermediate cross-state transitions. Moreover, noise-induced barrier crossing may lead to coexistent E, M, and hybrid E/M phenotypes, as well as emergence of stem-like circulating tumor cells (CTCs), thereby causing collective dissemination of primary tumors (Kudo-Saito et al., 2009; Jolly et al., 2015; Grigore et al., 2016; Bocci et al., 2019a,b), variation in tumor-seeding abilities (Neelakantan et al., 2017; Grosse-Wilde et al., 2018), and differences in drug sensitivity (Creighton et al., 2009; He et al., 2019; Tièche et al., 2019). Therefore, drug resistance, full and partial EMT (Aiello et al., 2018), and metastasis may all have underlying threshold-crossing mechanisms through multistability (Lee et al., 2014; Li and Balazsi, 2018).

Ultrasensitivity is the second threshold-generating property related to sharp input–output transfer functions with switch-like characteristics (Ferrell and Ha, 2014) in monostable systems. Monostable cells are those for which mathematical models predict a single steady state. From an experimental perspective, monostable cells return to their original state (protein and mRNA levels) upon a temporary perturbation, even if they are highly sensitive and thus deviate far. Ultrasensitivity leads to abrupt, large cellular responses to small, persistent input differences within a narrow input range (Figure 1B). For example, monostable cells can be ultrasensitive when their response to an internal or external factor is sigmoidal. For a system exhibiting ultrasensitivity, a threshold can be defined as the stimulus level (i.e., metabolite, protein, or cofactor concentration) at which the system is maximally sensitive (Louis and Becskei, 2002; Zhang et al., 2013).

The last mechanism of threshold generation is irreversibility, whereby external factors restrict or block the reversion of cellular processes, as in embryonic development (Caplan and Ordahl, 1978). Both monostable and multistable cells may approach physical or biochemical barriers that, once crossed, prevent reversion causing permanent outcomes. Such irreversibility can stabilize new cellular states. Typical examples are commencement of DNA synthesis, cell lysis, and apoptosis, which, after a certain progression, cannot revert (Figure 1C). Likewise, when a tumor cell enters the bloodstream and travels away, it is very unlikely to return to its original site (Scott et al., 2013), and even if it does, it will be already altered due to its time in a different environment.

Each of these three threshold-generating mechanisms can produce biological consequences in populations of cells ranging from cell division and neuron depolarization to apoptosis when a certain threshold is exceeded (Mateo et al., 2011; Xie et al., 2011; Sato et al., 2013). A common conceptual way to connect cell population phenotypes, such as drug resistance, oncogenesis, and metastasis to single-cell behaviors is through noise-modulated threshold crossing. However, altering the mean can also move cell populations closer to or farther from thresholds without any change in the noise. Therefore, investigating the role of noise in such processes requires fixing the mean. Yet, in natural systems, the mean and noise of protein levels have a tendency to be coupled (Newman et al., 2006; Dar et al., 2016), where higher means often associate with lower noise, along a hyperbolic (CV^2^ = *c*/mean) interdependence. Typically, this relationship holds for various bursting regimes (*c* > 1 in Figure 1D). From an observational standpoint, naturally occurring mutations or other changes make it rarely possible (Dar et al., 2014; You et al., 2019) to parse out specifically how the noise of a single protein affects threshold crossing and phenotypes independently of the mean. For example, Figure 1E shows the threshold levels that a given protein can naturally overcome based on the experimentally demonstrated inverse relationship (Newman et al., 2006; Dar et al., 2016) between its expression mean and noise (Figure 1D). Overall, due to their natural coupling tendency, both the mean and noise will change and affect threshold crossing. To decipher their individual impact on biological phenotypes, we need engineering approaches to independently control the protein noise and the mean because observing natural decoupling scenarios is far from trivial. In the next section, we explore ways of decoupling means and noise, with implications on drug resistance and metastasis.

## Controlling Non-Genetic Heterogeneity of Metastasis Through Synthetic Biological Gene Circuits

To confirm observational suggestions on the role of non-genetic heterogeneity in disease development, stress survival, and metastasis, one must control protein noise independently of the mean in living organisms. Engineering approaches from the field of synthetic biology (Elowitz and Leibler, 2000; Gardner et al., 2000) enabled the inception of such control (Blake et al., 2006; Fraser et al., 2009), followed by identification of many methods that reduce or amplify gene expression noise (Maamar et al., 2007; Cagatay et al., 2009; Nevozhay et al., 2013; Shimoga et al., 2013; Farquhar et al., 2019; Guinn and Balázsi, 2019). In natural systems, a change in a protein's mean will often change the noise since the two parameters tend to be coupled (Figure 1D). Therefore, biological threshold crossing typically does not utilize the mean and noise as two fully independent degrees of freedom (Figure 1E). As opposed to natural mechanisms, synthetic biological systems can allow independent changes in the mean and noise such that they are no longer coupled. Synthetic gene circuits thus provide an increasing number of ways to allow noise-mean decoupling (Aranda-Díaz et al., 2017; Farquhar et al., 2019) in studies of noise-modulated phenotypic transitions.

Two main approaches have accomplished decoupling the gene expression mean and noise from one another in synthetic gene circuits: (i) different gene circuits to express the same gene with different noise-vs.-mean dependencies (Blake et al., 2006; Süel et al., 2006; Kim and Sauro, 2010; Farquhar et al., 2019) and (ii) combinatorial induction of cascaded modules within the same gene circuit (Aranda-Díaz et al., 2017). Decoupling the noise and mean from one another adds a new degree of freedom to tune threshold crossing and reveal individual contributions of mean and noise on cellular processes, such as metastasis.

As an illustrative classification inspired by studies examining how mean and noise affect drug resistance (Blake et al., 2006; Farquhar et al., 2019), we consider a threshold for a metastasis activator (or a threshold for a metastasis inhibitor). Bistability, ultrasensitivity, and irreversibility underlying such thresholds are frequent themes in metastasis and EMT (Lee et al., 2014; Zhang et al., 2014; Jolly et al., 2015). Based on the position of the threshold (above or below) relative to the mean, and the ability to tune the mean and variance independently up and down, there are 16 possible phenotypic scenarios (Figure 1F). To start, assume metastasis activator levels are below a phenotypic threshold when the cells are not metastatic (Figure 1F, 1–4). In such a scenario, both the mean and variance of activator levels can be tuned up or down. Two of these changes (tuning mean or variance up) should promote metastasis by enhanced threshold crossing, while the two opposite changes (tuning mean or variance down) should hinder metastasis. Alternatively, for a metastatic cell population with hyperthreshold activator distribution (Figure 1F, 5–8), tuning the mean down or variance up should hinder metastasis, while the opposite changes should promote metastasis. Overall, for underthreshold populations, elevated activator mean and variance consistently aid overcoming the threshold. In contrast, for hyperthreshold populations, elevated activator variance still aids, but elevated mean hinders threshold crossing. Analogously, non-metastatic and metastatic cell populations could also have metastasis inhibitor levels above or below a phenotypic threshold, respectively (Figure 1F, 9–16). Tuning the variance and mean of a metastasis suppressor up or down will affect threshold crossing according to the above principles but with opposite phenotypic effects. Similar reasoning can predict the effect of simultaneous changes in the mean and the noise, although the scenarios can be numerous and complicated.

Protein mean- and noise-dependent crossings of various thresholds in synthetic and natural scenarios can also be explored visually as landscapes (Figure 1G). Synthetic biological control can explore surfaces with two degrees of freedom, whereas natural coupling restricts movement to a single degree of freedom along paths on such surfaces. These landscapes and curves illustrate what phenotypic threshold levels can be crossed when the noise and mean are coupled or uncoupled, respectively. In the future, it will be important to examine similar but multimolecular threshold crossings in higher dimensions, or the joint effects of the mean, the noise, as well as of the higher moments (i.e., skewness, kurtosis, etc.). Moreover, specific fitness landscapes (Nevozhay et al., 2012; González et al., 2015) will need to replace threshold approximations in many realistic scenarios. Synthetic biological tools will be indispensable for addressing these future questions.

Experimental evidence for how gene expression mean and noise independently affect metastasis threshold crossing is relatively lacking, but synthetic biology is already shedding light on drug resistance (Farquhar et al., 2019), which may give insights to the role of noise in metastasis as an evolutionary process. According to the threshold-crossing principles, high noise aids drug resistance evolution when stress is high but hampers survival when stress is low, mimicking the effects of noise on short-term survival (Blake et al., 2006; Fraser et al., 2009; Farquhar et al., 2019). Analogously, high noise should facilitate metastasis initiation for pre-metastatic cells before dissemination. In contrast, high noise may hinder the rate of metastasis for cells that have already acquired invasive characteristics. These findings relate to where cells sit (below or above) relative to thresholds at which specific phenotypes emerge. We anticipate that utilizing noise amplifying or reducing gene circuits (Becskei et al., 2001; Hooshangi et al., 2005; Weinberger et al., 2005; Tan et al., 2009; Diao et al., 2016; Farquhar et al., 2019) will give similar insights into processes underlying metastasis. Using the growing repertoire of noise-controlling synthetic biology tools and chemicals will certainly uncover unknown roles of gene expression in processes of full or partial EMT (Aiello et al., 2018), metastasis, and oncogenesis.

## Author Contributions

MG, YW, and GB conceived and wrote the manuscript. SL surveyed the literature and added the references. DY and MR contributed some critical concepts and feedback on the manuscript. All authors contributed to the article and approved the submitted version.

## Conflict of Interest

The authors declare that the research was conducted in the absence of any commercial or financial relationships that could be construed as a potential conflict of interest.
